# Metabonomics Research Progress on Liver Diseases

**DOI:** 10.1155/2017/8467192

**Published:** 2017-02-21

**Authors:** Mengqian Yu, Ying Zhu, Qingwei Cong, Chunyan Wu

**Affiliations:** Department of Infectious Diseases, The First Affiliated Hospital of Dalian Medical University, Dalian, Liaoning 116000, China

## Abstract

Metabolomics as the new omics technique develops after genomics, transcriptomics, and proteomics and has rapid development at present. Liver diseases are worldwide public health problems. In China, chronic hepatitis B and its secondary diseases are the common liver diseases. They can be diagnosed by the combination of history, virology, liver function, and medical imaging. However, some patients seldom have relevant physical examination, so the diagnosis may be delayed. Many other liver diseases, such as drug-induced liver injury (DILI), alcoholic liver disease (ALD) and nonalcoholic fatty liver disease (NAFLD), and autoimmune liver diseases, still do not have definite diagnostic markers; the diagnosis consists of history, medical imaging, and the relevant score. As a result, the clinical work becomes very complex. So it has broad prospects to explore the specific and sensitive biomarkers of liver diseases with metabolomics. In this paper, there are several summaries which are related to the current research progress and application of metabolomics on biomarkers of liver diseases.

## 1. Introduction

Metabolomics is a new discipline of the “postgenomics” period, which constitutes the core of biology systems with genomics, transcriptomics, and proteomics together and is one of the world's most active fields of life science research. The definition of metabonomics was firstly proposed by the Imperial College London, UK, Dr. Nicholson in 1999 [1]. It is a technique by examining the dynamic changes of metabolites to study metabolomic networks of biological systems before and after stimulations or disturbances (such as a specific genetic mutation or environmental change). The object of metabolomics study is endogenous small molecule, of which the relative molecular mass is less than 1000.

Metabolomics has the following advantages [2]. (1) Small changes in gene and protein expression will be reflected and amplified by the metabolite. (2) Study of metabonomics will not need full genome sequencing and a large number of expressed sequence tags (EST). (3) Metabolites are far less than the number of genes and proteins. (4) The physiological or pathological condition of the body can be detected through the analysis of metabolites in biological fluids. (5) As the detected metabolites in different organisms are similar, the use of metabolomics technique is more common.

The commonly used analysis techniques of metabonomics include nuclear magnetic resonance (NMR), gas chromatography-mass spectrometry (GC-MS), and liquid chromatography-mass spectrometry (LC-MS).

The NMR is one of the earliest and most common used techniques [3]. The spectroscopic technique is based on a spin nuclear property that absorbs radiation at the external magnetic field to generate nuclear energy transitions. The advantages of NMR include the demand for small samples, no sample pretreatment, and nondestructive and noninvasive detection. Furthermore, it can obtain the NMR spectrum of a specific living body portion noninvasively and quickly. NMR is the only existing metabolomics analysis technique which can be used in vivo and in situ studies [4].

Currently the most widely used and most effective metabolomics technologies are GC-MS [5] and LC-MS [6]. The former is appropriate to analyze small molecules, thermally stable, volatile, and easily gasified compounds and the latter can analyze the compounds more polar, higher relative molecular mass and lower thermal stability. In clinical studies, due to the use of blood, urine, and tissue samples, these samples are rich in various polar, nonvolatile, and nonvaporized substances, so compared with GC-MS, the applications of LC-MS are more widely used.

The common pattern recognition methods of metabolomics include unsupervised and supervised ones. The unsupervised methods contain principal component analysis (PCA) [7], nonlinear mapping (NLM) [8], hierarchical cluster analysis (HCA) [9], and so on, while the supervised methods include partial least squares-discriminant analysis (PLS-DA) [10] and artificial neural networks (ANN) [11]. The PCA and PLS-DA are the most commonly used methods of pattern recognition.

## 2. The Application of Metabonomics in Liver Disease

Liver diseases are the worldwide public health problems. Many of them, such as DILI, ALD, NAFLD, and autoimmune liver diseases, lack specific clinical diagnosis markers and the diagnoses consist of history and relevant score, so they have the high subjectivity, inaccuracy, and limitation. With the development of metabolomics, they have become a hot research field and have the broad prospect to explore the sensitive and specific liver diseases' biomarkers.

### 2.1. Metabonomic Studies on DILI

In the United States, drug-induced acute liver failure accounted for over 50% of all acute liver failure [12]. In China, DILI accounted for about 10% of patients hospitalized with acute hepatitis [13]. Mild DILI only showed elevated transaminase; the severe one might cause liver failure leading to death [14]. Due to the lack of specific and sensitive diagnostic markers of DILI, it is difficult to make early diagnosis and treatment leading to poor prognosis.

Now there are several clinical and experimental metabolomic studies on DILI trying to find the specific and sensitive markers. The main information about those researches is shown in Table 1.

As mentioned in Table 1, these metabolites were involved in carbohydrates, lipids, amino acids, bile acids metabolism, and inflammatory response. Although the results varied, they provided the preliminary understanding on the pathophysiological process of DILI and were beneficial for the further study of DILI markers.

### 2.2. Metabonomics Studies on ALD

Alcoholic liver disease is divided into alcoholic fatty liver, alcoholic hepatitis, alcoholic liver fibrosis, alcoholic liver cirrhosis, and even alcoholic liver cancer. The diagnosis consists of history of drinking, liver function, medical imaging, and clinical symptoms. In general, alcoholic liver disease has no obvious symptoms and most patients will not go for physical examination. When the disease was found, it might have been in advanced stage. In order to find sensitive and specific biomarkers and make early diagnosis, there were several clinical and experimental metabolomic studies on ALD. The main findings are shown in Table 2.

As mentioned in Table 2, there were two LC-MS based urinary metabolomic studies which both showed the correlation between indole-3-lactic acid and ALD. This might mean that indole-3-lactic acid could be used as a potential diagnostic marker of ALD.

### 2.3. Metabonomics Studies on NAFLD

NAFLD ranges from simple nonalcoholic fatty liver (NAFL) and nonalcoholic steatohepatitis (NASH) to nonalcoholic cirrhosis. Although NAFL is a benign process and can be reversed through diet and exercise, it has the risk of progressing to NASH and nonalcoholic cirrhosis and causing type 2 diabetes (T2MD) [24]. NAFLD lacks sensitive and specific clinic diagnostic biomarkers. With the development of metabolomics, it provides a powerful tool for NAFLD research.

Now there are several clinical and experimental NAFLD metabolomic researches. However, there are still no uniform metabolites which could be used as the diagnostic markers of NAFLD. The details are shown in Table 3.

### 2.4. Metabonomics Studies of Hepatitis Virus Related Liver Diseases

According to the World Health Organization, about 2 billion people worldwide have been infected with HBV, of which 240 million people are chronically infected with HBV and each year about 650 thousand people died of liver failure, cirrhosis, and hepatocellular carcinoma caused by HBV.

#### 2.4.1. Metabonomics Studies on Chronic Hepatitis B

Natural history of chronic hepatitis B infection is generally divided into immune tolerance, immune clearance, inactivation, and reactivation. The immune clearance phase is the best time of antiviral therapy. The clinical diagnosis requires a combination of virology, liver enzyme, prothrombin activity (PTA), history, and medical imaging to make a comprehensive assessment, which results the clinical work tedious, so it is of great significance to explore new sensitive and specific biomarkers to attain efficient antiviral treatment and good prognosis.

At present, several metabolomic researches on chronic hepatitis B (CHB) have been made. However, there were no uniform biomarkers of CHB, so it might be necessary to make larger number of clinical studies. Now the relevant researches are shown as in Table 4.

#### 2.4.2. Metabonomics Studies on Hepatitis B Virus-Induced Liver Cirrhosis

Hepatitis B virus- (HBV-) induced liver cirrhosis is often secondary to CHB. When it is found, liver usually has irreversible change and the prognosis is poor, so the detection of sensitive and specific biomarkers of early fibrosis has important clinical significance.

Till now, some researchers have made several studies on HBV-induced cirrhosis trying to find the new sensitive and specific biomarkers. However, due to some limitations, those results still could not be used as biomarkers of HBV-induced cirrhosis.  Table 5 shows the contents of relevant researches.

#### 2.4.3. Metabonomics Studies on HBV-Induced Liver Failure

Liver failure is a severe liver disease which progresses rapidly and has high mortality and poor prognosis and lacks the sensitive and specific biomarkers. We diagnose it by the combination of clinical symptoms, liver function, prothrombin activity (PTA), and medical imaging. Due to the lack of sensitive and specific markers, when liver failure was found, it might have been in progress, resulting in poor prognosis.

In China, liver failure is divided into four types: acute liver failure, subacute liver failure, acute-on-chronic liver failure, and chronic liver failure. The type determines the severity and prognosis of the disease. We usually distinguish the type by a detailed history; it means that there is certain subjectivity and limitation. So it has very important significance to detect sensitive and specific biomarkers of liver failure. At present, there are some scholars who have made some research trying to find the biomarkers. The main results will be shown in Table 6.

As shown in Table 6, the main metabolites were involved in lipid metabolism disorders. It has been reported that LPCs regulate cell proliferation, tumor cell invasiveness, and inflammation and play an important role in immunity adjustment. It is helpful to further understand the pathogenesis of liver failure. However, as the current researches are limited, the extensive researches are needed to verify these.

#### 2.4.4. Metabonomics Studies on HBV Induced Hepatocellular Carcinoma

Hepatocellular carcinoma (HCC) is the fifth malignancy worldwide and is the third leading cause of cancer-related deaths worldwide. The diagnosis of HCC usually combines alpha-fetoprotein (AFP) with medical imaging. However, the specificity of AFP is poor and AFP can also increase in severe hepatitis, ovarian tumors, and embryonal tumors. Liver biopsy is the gold standard for the final diagnosis of HCC; however it is an invasive diagnostic method and brings great suffering to patients, so it is difficult to be accepted by patients. Therefore it is of importance to explore the sensitive and specific diagnostic markers.

At present, several metabolomic studies have been made to find the more sensitive and specific markers.  Table 7 shows the main contents of these researches.

As shown in Table 7, the representative metabolic disorders included lipid metabolism, bile acid metabolism and amino acid metabolism. LPCs were the most significant changes among lipid metabolism. It has been reported that LPCs regulate cell proliferation, tumor cell invasiveness, and inflammation and play an important role in immunity adjustment. Some researchers have proved that bile acids play an important role in lipid and glucose metabolism, energy consumption, and even liver regeneration. The main changed amino acids in Table 7 have some functions, including providing one-carbon unit, as raw material of gluconeogenesis and antitumor immune response. These metabolites are helpful to further understand the pathogenesis of liver cancer. However, the metabolites as diagnostic biomarkers have not reached the consensus, so further studies might be needed.

### 2.5. Metabonomics Studies on Autoimmune Liver Diseases

Autoimmune liver diseases include autoimmune hepatitis (AIH), primary biliary cholangitis (PBC), and primary sclerosing cholangitis (PSC). The clinical diagnosis depends on liver-related antibodies, liver function, liver histology, and the excluding of hepatitis virus, drug, and alcohol to make the comprehensive score. However, because some items of the score system cannot be achieved, the accuracy decreases. Therefore, it is very necessary to explore the sensitive and specific biomarkers of autoimmune liver diseases.

Till now, there have been several researches about autoimmune liver diseases. However, there are no unified results because of the different methods and limited number. Now we will show the relevant researches results in Table 8.

## 3. Discussion

The main metabolites mentioned in Table 8 represented several metabolic disorders, including glucose metabolism, especially the citric acid cycle, lipid metabolism, amino acid metabolism, urea cycle, bile acid metabolism, bilirubin metabolism, gluconeogenesis, inflammation, immune response, cell regeneration, and apoptosis. These metabolisms interact with each other in liver disease and promote the development of liver disease together.

Among these metabolites, we found that PC and LPC decreased in all liver diseases and some studies found that FA reduced. At present, some studies have shown that PC is involved in lipoprotein composition [56] and plays an important role in the maintenance of cell membrane function [57]. PC could decompose into choline and LPC; choline could promote the synthesis of GSH which played the role of antioxidant [57]. In liver diseases, hepatocytes suffered an injury and oxygen free radicals increased. PC participated in the repair of cell membranes and promoted the generation of choline to synthesize GSH acting as antioxidant, so PC showed the downtrend and its degradation products showed the same trend. Some studies have found that FA is the major energy resource under pressure [58]. It could provide energy for hepatocyte to facilitate its function by *β*-oxidation. Moreover, FA could be converted to ketones and glucose. In liver diseases, the citric acid cycle was blocked; FA broke down to produce energy, so it could explain the decreased level of FA.

In some researches above, we can see in liver diseases, except for liver cancer, that glucose, lactic acid, and citric acid increased. Glucose and citric acid may increase with the disorder of citric acid cycle. When hepatocyte was injured, it caused the mitochondrial dysfunction, resulting in the disorder of citric acid cycle. Furthermore, the increase of glucose was accompanied by the decreased glycerol and some amino acids such as glutamate, glutamine, and valine. These substances could be converted to glucose through gluconeogenesis and then enter the glucose metabolic pathways to produce energy. In liver diseases, the aerobic metabolism was blocked; glycolysis and pentose phosphate pathway became the main glucose metabolic pathways. Glycolysis could produce large amounts of lactic acid, leading to the high lactic acid level [59, 60].

Amino acids can be divided into glucogenic amino acids, ketogenic amino acids, and glucogenic and ketogenic amino acids. In liver diseases, the aerobic metabolism was injured; glycolysis became mainly glucose metabolic pathways, producing less ATP and arousing gluconeogenesis. Glucogenic amino acids such as glutamic acid and glutamine converted into glucose, leading to the decrease of amino acids. In patients with cirrhosis, the branched-chain amino acid decreased, while the aromatic amino acids increased; the ratio of branched-chain amino acid and aromatic amino acid was out of balance. In addition, some studies have shown that high levels of aromatic amino acids, especially phenylalanine, are related to the impact of the gut microorganisms [61, 62].

The most metabolites declined in HCC, but the levels of glutamine and malic acid increased; this might be correlated with urea cycle disorders. When liver cells lost their normal function and urea cycle was injured, causing elevated blood ammonia. Glutamine as the detoxification products showed the elevated levels. Malic acid could be produced by fumaric acid and generate oxaloacetate which could promote the formation of aspartic acid in transamination process and aspartic acid was used in urea cycle. When urea cycle was disordered, aspartic acid consumption reduced, indirectly leading to malic acid accumulation.

Besides the metabolic pathways above, other metabolic pathways also involved bile acid metabolism and bilirubin metabolism. Bile acids synthesized in hepatocyte cytoplasm and microsomes and excreted into intestine through the bile duct. When liver cells were damaged, the bile acids in cytoplasm were released into blood, causing the elevated serum bile acid levels. Conjugated bilirubin came into being by unconjugated bilirubin in smooth endoplasmic reticulum of liver cells. When hepatocytes or bile duct cells swell, degeneration and necrosis and the secretion and excretion of bilirubin were in disorder, leading to the release and regurgitation of bilirubin into blood and resulting in the high serum bilirubin level.

## 4. Conclusion and Outlook

To date, metabolomics has had considerable progression, but as a novel technology, it also faces the challenges of methodology and application. From the methodological point, the current data analysis strongly depends on the relevant technical expertise and experience; the existed analysis instruments and techniques or data processing methods all need further development. From the application point, many liver-related metabolomics researches could only recognize the different metabolites between each group, while these metabolites had not been used as sensitive and specific markers for the diagnosis of diseases. Although metabonomics has some deficiency as mentioned above and is at the early stage of development, metabonomics application in liver disease has become a hot topic in the academic field. We believe that, with the further technological innovation, the application of metabolomics in the field of liver disease will have broad prospects in the aspects of finding the new early specific markers of different liver diseases and different stages of the same disease and providing new objective evidences for the diagnosis, treatment, and prognosis.

## Figures and Tables

**Table 1:** The main information about clinical and experimental metabolomic studies on DILI.

Reference	Methods	Sample	Main findings
[15]	UPLC-TOF-MS	Serum	LPC 16:0, LPC 18:0, LPC 18:2, and LPC 18:3↓; glycylchenodeoxycholic acid, glycocholate, bilirubin, stearic acid amide, oleic acid amide, myristyl amide, and hypoxanthine↑

[16]	^1^H-NMR	Serum and urine	Serum metabolites: lactate, glucose, 3-hydroxyisovalerate, isoleucine, acetylglycine, acetone, acetate, glutamine, ethanol, and isobutyrate↑; urine metabolites: citrate, glycine, hippurate↓; 3-chlorotyrosine, phenylalanine, and glutarate↑

[17]	UPLC-TOF-MS/MS	Serum	sn-1 monoacylglycerophosphocholine, sn-2 arachidonyl diacylglycerophosphocholine, and sphingolipids↓; diacylglycerophosphocholines, monoacylglycerophosphoethanolamines, and amino acids↑

[18]	GC-MS	Serum	NEFAs: C18:1n9, C18:1n7, C18:2n6, C20:3n6, C20:4n6, C20:5n3, and C22:6n3↑; all EFAs except C12:0↑

UPLC-TOF-MS: ultraperformance liquid chromatography/time-of-flight mass spectrometry.

UPLC-TOF-MS/MS: ultraperformance liquid chromatography/time-of-flight tandem mass spectrometry.

^1^H-NMR: a proton nuclear magnetic resonance.

GC-MS: gas chromatography-mass spectrometry.

**Table 2:** The main results of clinical and experimental metabolomic studies on ALD.

Reference	Methods	Sample	Main findings
[19]	CE-TOF-MS	Serum	19 metabolites associated with alcohol intake, and three biomarker candidates (threonine, guanidinosuccinate, and glutamine) were related to alcohol-induced liver injury. Glutamate/glutamine ratio might also be good biomarker.

[20]	HPLC-ion trap-MS	Urine	Seven metabolites were identified, including creatinine, indole-3-carboxylic acid, indole-3-lactic acid, L-tryptophan, L-serine, L-leucine, and glutathione.

[21]	^1^H-NMR	Serum	Metabolites significantly increased with large HCC were glutamate, acetate, and N-acetyl glycoproteins; metabolites that correlated with cirrhosis were lipids and glutamine. Metabolomic profiles of small HCC patients were similar to those of large HCC group.

[22]	UPLC-ESI-QTOF-MS	Urine	Indole-3-lactic acid and phenyllactic acid may serve as robust noninvasive biomarkers for early stages of ALD.

[23]	UPLC-QTOF-MS	Serum	Five metabolic pathways were identified, namely, phenylalanine and tyrosine metabolism, leucine degradation, tryptophan metabolism, sphingolipid metabolism, and glycerophospholipid metabolism. Serum LPCs showed disease-specific changes, probably reflecting metabolic difference between liver injury and HCCX in nude mice.

CE-TOF-MS: capillary electrophoresis time-of-flight mass spectrometry.

UPLC-ESI-QTOF-MS: ultraperformance liquid chromatography coupled with electrospray ionization quadrupole time-of-flight mass spectrometry.

**Table 3:** The findings of metabolomic researches on NAFLD.

Reference	Methods	Sample	Main findings
[25]	LC-QTOF-MS and GC-QTOF-MS	Serum	*α*-Ketoglutarate levels are significantly increased in obese patients compared with lean controls.

[26]	UPLC-MS/MS	Serum and urine	Serum and urine DCA concentrations were statistically significantly increased. Patients with NASH exhibited a greater postprandial increase in all bile acid groups except LCA and 6*α*-hydroxylated bile acids. Patients with NASH had decreased glycine- and taurine-conjugated LCA exposure over the postprandial period.

[27]	CE-TOF-MS and LC-TOF-MS	Serum	Etiocholanolone-S, DHEA-2S, and DHEA-S decreased with the progression of fibrosis, while 16-OH-DHEA-S increased with the progression of fibrosis. The ratio of 16-OH-DHEA-S to DHEA-S (16/D) and the ratio of 16-OH-DHEA-S to etiocholanolone-S (16/E) were more clearly associated with the fibrosis grade.

[28]	UPLC-QTOF-MS/MS	Serum	Strong overlap of the animal model samples and human NAFLD patients is observed in the sn-1 monoacylglycerophosphocholine profile. Seven sphingomyelin type lipids: (SM 36:3), (d18:2/16:0), (d18:2/14:0), (d18:1/18:0), (d18:1/16:0), (d18:1/12:0), and (d18:0/16:0) were found to be significantly altered in the human NAFLD patients compared to normal liver subjects; similar tendencies were also found in the animal model samples. Deoxycholic acid was found significantly higher both in the animal model samples and in the human NAFLD patients.

[29]	GC-MS	Tissue	Hydroquinone (HQ) and nicotinic acid (NA) suggest a protective effect against NAFLD. However, only NA showed marked effects on both steatosis and transaminase levels suggesting its potential as a therapeutic or preventive agent in NALFD.

[30]	HPLC-QTOF-MS	Tissue and serum	Stearoylcarnitine increased notably in both rat liver tissue and serum. However, the potential as biomarker of NAFLD still needs to be confirmed by extensive studies.

[31]	^1^H-NMR	Serum	Four potential biomarkers for diagnosis of NAFLD stages were selected: serum glucose, lactate, glutamate/glutamine, and taurine. A specific combination of spectroscopic changes in glucose, lactate, glutamate/glutamine, and taurine levels may prove to be an accurate means of noninvasively diagnosing various stages of NAFLD.

**Table 4:** The relevant information about researches on CHB.

Reference	Methods	Sample	Main findings
[32]	UPLC-TripleTOF-MS/MS	Serum	Lysophosphatidylcholine, 3-oxodecanoic acid, and bile acids (TCA, GCA, and TCDCA) had high diagnostic value on immune clearance; oleamide amide was elevated in the three groups of CHB but was more notable in the inactive phase and had high diagnostic value on inactive phase.

[33]	UPLC-QTOF-MS	Urine	Biotin sulfone, 5-oxo-heneicosanoic acid, d-Glucosaminide, and 2-methylhippuric acid were the most significant differential metabolites for the classification of the HBV and the control.

[34]	GC-TOF-MS	Serum	Citric acid, aconitic acid, glutamine, N,N-dimethyl glycine, and malonic acid had good correlation with hepatitis B and had high sensitivity and specificity to differentiate CHB from control.

[35]	^1^H-NMR	Cell fluid	Uridine, inosine, guanosine, uracil, and xanthine were all significantly decreased.

**Table 5:** The main contents of metabolomic studies on HBV-induced cirrhosis.

Reference	Methods	Sample	Main findings
[36]	UPLC-LTQOrbitrap-MS	Serum	The nine metabolites differentiated A-grade from the control group, including nicotinamid e, aminoadipic acid, glutamine, tyramine, dodecenoic acid, lysophosphatidylcholine, glycol-cysteine, cysteine amino acid, and octenoic acid; three metabolites that distinguished grade A from B, including ethanolamine, glycine, glycosylchenodeoxycholic acid; 10 metabolites that differentiated grade B from C, including aminoadipic acid, taurine, aminoacetone, glycine, pyruvate, glycolcholodeoxycholic acid, alanine, pipecolic acid, methionine, and serine.

[37]	RP-HPLC-QTOF-MS and HILIC-QTOF-MS	Serum	Oleic acid, bilirubin, acetylcarnitine, and GCDCA were significantly increased in cirrhosis patients and distinguished cirrhosis from control.

[38]	GC/MS	Serum	Acetic acid, sorbitol, D-lactic acid, hexanoic acid, 1-naph-thalenamine, butanoic acid, phosphoric acid, D-glucitol, and glucose were the strongest segregation between cirrhosis and CHB.

[39]	^1^H-NMR	Serum	Compared with the compensatory period, some metabolites increased significantly, including glucose, citrate, succinate, phenylalanine, tyrosine, lysine, glutamine, and creatine, whereas some decreased notably, namely, LDL, VLDL, N-acyl glycoprotein (NAG), choline, acetone, isoleucine, and valine in the decompensation period.

UPLC-LTQOrbitrap-MS: ultraperformance liquid chromatography coupled with linear trap quadrupole Orbitrap mass spectrometry.

RP-HPLC-QTOF-MS and HILIC-QTOF-MS: reversed-phased (RP) high liquid chromatography and hydrophilic interaction chromatography (HILIC) coupled with quadrupole time-of-flight mass spectrometry.

**Table 6:** The main findings of studies on liver failure.

Reference	Methods	Sample	Main findings
[40]	UPLC-LTQ Orbitrap-MS	Serum	LysoPC(18:0), lysoPC(17:0), lysoPC(16:0), lysoPC(15:0), PA(20:4(5Z,8Z,11Z,14Z)e/2:0), phenylalanyl phenylalanine, bilirubin glucuronide, acetoacetic acid, L-threonine, and DHAP(18:0) showed significant differences between the survival group and nonsurvival group; lysoPC(14:0), phenylalanyl phenylalanine, and bilirubin glucuronide improved with treatment and had potential disease-monitoring capability.

[41]	UPLC-QTOF-MS	Serum	Phosphatidylcholine, lysophosphatidylcholine, nonconjugated bile acids, and conjugated bile acids were considered as common biomarkers of ACLF and CLF group, while linoleyl carnitine showed significant increase in CLF compared with ACLF; it was considered as the differential marker of the diagnosis of CLF and ACLF.

[42]	HPLC-ion trapTOF-MS	Serum	1-Linoleoylglycerophosphocholine or 1-linoleoylphosphatidyl choline was found significantly different between healthy controls and liver failure group.

**Table 7:** The main results of relevant metabolomic studies on HCC.

Reference	Methods	Sample	Main findings
[43]	RRLC-QTOF-MS	Serum	Tryptophan decreased in the sera of CHB, CIR, and HCC patients; C16:1-CN, as one of the long-chain acylcarnitines, increased with severity of chronic liver diseases.

[44]	UPLC-linear ion trap Q-Orbitrap-MS	Urine	Adenosine, inosine, cyclic AMP, and citric acid increased significantly, while xanthine, MTA, 6-methyladenosinie, CA, GCA, and GCDCS decreased notably in liver diseases than healthy control group. Carnitine C4:0 and hydantoin-5-propionic acid were defined as a combinational marker to distinguish HCC from CIR.

[45]	UPLC-QTOF-MS	Urine	Palmitic acid, alpha-N-phenylacetyl-L-glutamine, phytosphingosine, indoleacetyl glutamine, and glycocholic acid were the most significant differential metabolites for the classification of the HCC and the control.

[46]	UHPLC-LTQ-Orbitrap-MS	Tissue and serum	In liver tissue, there were 880 metabolites that could differentiate hepatocellular carcinoma tissue from distal noncancerous tissue, while in serum, betaine and propionylcarnitine were selected as the optimal combination for diagnosis of hepatocellular carcinoma.

[47]	UPLC-QTOF-MS and UPLC-QqQLIT-MS and UPLC-triple quadrupole-MS	Serum	GCA, GDCA, TCA, and TCDCA were significantly downregulated in sera of HCC versus those with cirrhosis, while lysoPC 17:0 and S-1-P showed a marginally significant upregulation.

[48]	GC-TOF-MS UPLC-QTOF-MS	Serum and urine	Five differential metabolites are found both in serum and urine samples in HCC patients, including glycocholic acid, cysteine, cystine, taurine, and phenylalanine. These metabolites represent bile acid metabolism, methionine metabolism, and phenylalanine and tyrosine metabolism.

[49]	UPLC-LTQ-Orbitrap-MS	Tissue	Betasitosterol, L-phenylalanine, lysoPCs, glycerol-phosphocholine, lysoPEs, enodeoxycholic acid glycine conjugate, and quinaldic acid were significantly lower in central tumor tissue group compared with the distant tissue group. Arachidyl carnitine, tetradecanal, and oleamide were significantly high in the central tumor tissue group compared with the distant tissue group. A comparison of the levels of these metabolites in the adjacent tissue group showed relative closeness to those in the central tumor tissue group.

[50]	CE-TOF-MS	Serum	Four metabolites including tryptophan, glutamine, arginine, and 2-hydroxybutyric acid fulfilled the demand of small HCC discrimination. The combination of Trp, Gln, and 2-hydroxybutyric acid was better to establish the discrimination model for the validation set (including small HCC subjects).

[51]	^1^H-NMR	Serum	LDL, VLDL, choline, and acetoacetic acid decreased in liver cirrhosis and hepatocellular carcinoma patients, while the contents of glutamine, pyruvate, phenylalanine, and tyrosine increased. These metabolites could differentiate patients from healthy people, but liver cirrhosis and hepatocellular carcinoma partially overlap.

[52]	RRLC-QTOF-MS UPLC-LTQ-Orbitrap-MS	Serum	Six major clusters were observed, the representative characteristic metabolites were selected from each cluster including LPC 22:5, palmitoyl-L-carnitine, LPC 22:6, LPE 16:0, LPC O-16:0, and TCA. Three metabolites including LPC 22:5, LPE 16:0, and TCA were selected as candidate markers for the classification of HCC and chronic liver diseases.

RRLC-QTOF-MS: rapid-resolution liquid chromatography quadrupole time-of-flight mass spectrometry.

UPLC-QqQLIT-MS: ultraperformance liquid chromatography coupled with triple quadrupole linear ion trap mass spectrometry.

**Table 8:** The main results of studies on autoimmune liver diseases.

Reference	Methods	Sample	Main findings
[53]	UPLC-MS	Serum	Glycochenodeoxycholic acid, FFA, LPC-16:0, PC-16:0/16:0, and SM could be used to diagnose AIH and PBC accurately.

[54]	UPLC-MS/MS	Serum	LCA-S, TDCA, and GDCA were significantly different between PSC and PBC samples.

[55]	^1^H-NMR	Serum	Citrate, glutamine, acetone, pyruvate, *β*-hydroxyisobutyrate, acetoacetate, histidine, dimethylamine, and creatinine had a high diagnostic accuracy for the discrimination of AIH from PBC.
